# MetaphorPrompt2—A Structure and Function-Focused Approach for Extracting Causal Events from Biological Text

**DOI:** 10.34133/csbj.0126

**Published:** 2026-07-03

**Authors:** Parth Patel, Yu-Chiao Chiu, Yufei Huang, Jianqiu Zhang

**Affiliations:** ^1^ Department of Electrical and Computer Engineering, The University of Texas at San Antonio, San Antonio, TX, USA.; ^2^ Department of Medicine, UPMC Hillman Cancer Center, University of Pittsburgh Medical Center, Pittsburgh, PA, USA.

## Abstract

Extracting molecular regulatory pathways (MRPs) from biomedical literature is crucial for building knowledge graphs that represent disease mechanisms and molecular regulation. However, even advanced large language models (LLMs) using in-context learning often misinterpret complex domain-specific causal statements or omit intermediary steps, resulting in incomplete pathway representations. MetaphorPrompt2 is motivated by cognitive theories of structural mapping and causal event representation. It improves MRP extraction by emphasizing structural relations and functional roles of biological entities rather than relying on surface-level grammar. The system integrates 5 components that collectively reduce parsing complexity and mitigate error propagation. At one-shot in-context learning, the proposed method achieves a 31% improvement in edge prediction F1 over the no-metaphor baseline and a 6.5% to 12.2% improvement over a previous method across 3 datasets: reguloGPT, BioInfer, and ADE. The percentage of causal links where both nodes are missed is reduced from 7.7% in the previous method to 3.7% in MetaphorPrompt2. These results support improved causal triplet extraction for biomedical pathway construction and potentially downstream utility in hypothesis generation or drug repurposing, which remains to be evaluated.

## Introduction

The extraction of molecular regulatory pathways (MRPs) from biomedical literature is a cornerstone of modern biomedical informatics. MRPs, which describe the complex interactions between genes, proteins, and other molecular entities, are critical for representing disease mechanisms, drug actions, and predictive outcomes such as drug responses and disease progression. Curated databases such as Kyoto Encyclopedia of Genes and Genomes (KEGG) [1] catalog these pathways, providing structured resources for downstream analysis. By accurately extracting MRPs from literature, researchers can systematically build comprehensive knowledge graphs (KGs) [2] that integrate diverse biological data sources. The quality and completeness of MRP extraction directly determine the utility of resulting KGs for downstream applications.

At the heart of MRPs are causal links—cause-and-effect connections that define how molecular entities interact dynamically to produce biological outcomes. Traditional MRP extraction has relied on manual curation, which is time-consuming and labor-intensive and struggles to keep pace with the exponential growth of biomedical literature. To address this, automated natural language processing (NLP) methods have been developed, combining rule-based and machine-learning strategies to improve the extraction of biomedical knowledge from literature, as seen in databases like RepoDB, MSI, Hetionet, DrugMechDB, and INDRA [3]. These traditional methods face substantial limitations: They often skip intermediary nodes in causal chains, leading to incomplete pathway representations. Additionally, they struggle with semantic inconsistencies and the complex propositional structures inherent in biomedical texts [4,5]. These limitations highlight the need for more robust and scalable approaches to MRP extraction [6].

Recent advances in large language models (LLMs), such as GPT-4 [7], have revolutionized NLP. Also, several techniques such as zero-shot and few-shot in-context learning (ICL) combined with prompt engineering and chain-of-thought (CoT) [8,9] strategies, retrieval-augmented generation (RAG) [10], and tool use/function calling [11] have shown promise in extracting complex information from biomedical texts. For example, reguloGPT [12], a state-of-the-art method, uses CoT prompts to capture the hierarchical structure of MRPs and resolve semantic inconsistencies.

However, LLM-based approaches face substantial challenges when applied to MRP extraction. While they excel at processing everyday language, they often struggle with domain-specific terminology and complex causal relationships [13] in biomedical texts. Moreover, their reliance on statistical patterns rather than deep understanding can lead to errors in extracting intermediary nodes when inferring causal links [14]. These deficiencies result in misidentified entity relationships or omission of nuanced causal mechanisms that underline biological processes, producing incomplete MRPs that compromise the quality of downstream KG construction [12,15].

Here, we clarify the difference between MRP extraction and contemporary biomedical relation extraction including representative fine-tuned systems such as GenBEE [16], AutoBioKG [17], BioNExt [18], and JTIS [19]; RAG systems include SyRACT [20] and CUI-RAG-style approaches [21]; and structured-output/tool-use approaches include JSON-schema or function-calling extraction frameworks such as LLM-IE [22]. These methods are important related work, but most target flat entity–relation–entity extraction or operate with task-specific supervision. This paper instead targets direct MRP extraction, where direct causal links, intermediary mediators, and functional roles must be preserved in a sparse causal graph rather than recovered as a flat list of binary triples. Direct F1 comparison to those systems would therefore conflate task definition, supervision level, annotation scheme, output structure, and evaluation target. We cite these methods for positioning, and we view RAG-based grounding and provider-level structured-output enforcement as complementary extensions rather than direct substitutes.

To address the challenges of LLM-based approaches in MRP extraction, MetaphorPrompt [15] was proposed. Unlike previous LLM-based methods such as reguloGPT-CoT [12], which query language models about known gene–gene interactions, MetaphorPrompt leverages analogical reasoning to translate complex biological processes into familiar, real-world metaphors and improve extraction of causal relationships. For example, the biological process “Protein A increases Protein B by suppressing Protein C” can be mapped to the metaphor “John increases productivity by reducing distractions”.

This decomposition-based approach is grounded in grammatical categorization. It guides LLMs to process biomedical titles through sequential steps: identifying linguistic elements (nouns and verbs), extracting context via prepositional markers, and developing metaphors by constructing everyday analogies from these grammatical components. The model then maps scientific to metaphorical terms, detects direct and indirect noun–noun interactions to infer causal relationships, determines causal events by identifying agents, patients, and effects, and finally outputs all causal links as structured nodes and edges (node A, node B, action) in a summarized document.

This prior work suggested that metaphor-structured prompting can improve MRP extraction compared with standard ICL. It achieved a 13% performance gain with 1- to 3-shot ICL and outperformed reguloGPT-CoT [12] with 4-shot ICL in both edge recall and precision metrics [15].

The effectiveness of this method may lie in how metaphor-structured prompts constrain LLM outputs: By mapping unfamiliar biomedical interactions onto familiar causal schemas, the model is redirected toward relational reasoning over high-frequency general-domain patterns rather than domain-specific surface features. We treat metaphor as a structured prompting scaffold, not as direct evidence that the LLM internally performs analogical reasoning in the cognitive-science sense.

Despite these strengths, the method suggests substantial limitations when processing complex molecular relationships. Our comprehensive analysis of 400 biomedical titles reveals that this approach fails to accurately capture causal relationships in a substantial number of cases. The failures were particularly pronounced in handling N6-methyladenosine (m6A) modifications, with m6A-related errors accounting for 171 instances across node identification tasks. For instance, in titles containing phrases such as “m6A-dependent manner”, the prompt consistently misidentifies modifiers as primary agents, fundamentally misrepresenting the underlying biological mechanisms.

We further analyzed the prompt design of MetaphorPrompt and found that this grammar-first approach fails to adhere to the structure-mapping theory (SMT) [23] of analogical reasoning, which rests on the core principle of systematicity, emphasizing the maintenance of coherent relational systems so that analogies preserve the integrity of relationships among entities. Furthermore, the method fails to encode state and state-transition information in causal events, losing essential mechanistic detail, thereby obscuring the true causal dynamics of the biological event.

In this paper, we propose MetaphorPrompt2, which addresses limitations in structure mapping and state-transition encoding through a revised architecture that classifies named entities according to functional roles, enforces one-to-one correspondence between biological entities and roles, and performs interaction identification prior to metaphor generation. We use Gopnik and Wellman’s causal event representation as a structural template for Input-State -> Process -> Outcome-State encoding while not claiming that LLM processing parallels child causal learning [24].

Empirical evaluation suggests that this structure- and function-focused approach improves entity identification and relationship mapping. We evaluated the proposed method on 3 datasets: reguloGPT [12], a curated benchmark of biomedical paper titles focused on m6A regulatory pathways; BioInfer [25], a biomedical sentence-level relation corpus; and ADE [26], an adverse drug event relation corpus. At one-shot ICL on GPT-4o, the proposed method improves over MetaphorPrompt by 12.0 percentage points on reguloGPT, 12.2 percentage points on BioInfer, and 6.5 percentage points on ADE. The gains transfer to GPT-5.4, suggesting that the improvement reflects the prompt architecture rather than a specific GPT-4o artifact.

Relative to the MetaphorPrompt [15], this paper adds (a) substantially expanded theoretical grounding in structure-mapping theory and causal event representation Theory, (b) evaluation on 2 additional datasets (BioInfer and ADE) beyond reguloGPT, (c) a comprehensive single- and multi-component ablation study, (d) a component degradation analysis that replaces refined modules with their conference-version counterparts, (e) detailed MRP element-level performance analysis, (f) cross-backbone evaluation on biomedical domain-tuned LLMs (Llama3-OpenBioLLM-70B and BioMistral-7B) and a recent frontier model (GPT-5.4) to address the concern of a narrow testing base, (g) statistical reliability analysis through multi-run stability evaluation and bootstrap confidence intervals (CIs) on the reguloGPT benchmark, and (h) qualitive inspection of difference of generated metaphors.

## Methods

### Theoretical foundation of MetaphorPrompt2

The design of MetaphorPrompt2 is grounded in 2 cognitive theories. One is Gentner’s structure-mapping theory [23], which posits that effective analogical reasoning depends on systematic correspondence between relational structures rather than surface similarities. In the context of MRP extraction, this theory directly addresses a fundamental challenge: Biomedical texts contain complex causal relationships that are difficult for LLMs to parse due to domain-specific terminology and intricate syntactic structures.

SMT suggests that analogical reasoning succeeds when 3 key principles are satisfied: (a) systematicity: coherent systems of relations are preferred over isolated predicates, (b) one-to-one correspondence: each element in the source domain maps to exactly one element in the target domain, and (c) semantic similarity: predicates that are similar in semantics across domains are preferred. The proposed method operationalizes these principles through its component architecture:

• Systematicity principle: The proposed method implements systematic correspondence preservation through its direct interactions identification component, which extracts complete relational structures using dependency parsing before metaphorical mapping occurs, ensuring that complex cascades like **“**A regulates B through C**”** maintain their structural integrity throughout the analogical reasoning process.
• One-to-one correspondence principle: This requirement is addressed through functional-role classification during Named Entity Identification, assigning each biological entity to exactly one role within the causal framework, initiator (entities that initiate an action or process), mediator (mediating entities that initiate and receive actions), or outcome (outcomes of actions or processes), thereby preventing ambiguous mappings and constraining all subsequent processing steps to maintain consistent entity–role relationships
• Structural alignment principle: To implement relational focus, functional relationships are prioritized over surface features by extracting Direct Interactions before metaphor generation, ensuring that biological processes map to everyday scenarios with analogous causal structures rather than superficial similarities, thereby enabling more accurate structural correspondence between source and target domains.

In contrast, the previous method adopts a grammar-first approach that prioritizes grammatical roles (nouns, verbs, prepositions) over functional relationships, thereby violating the principle of systematicity by fragmenting coherent causal chains into isolated grammatical elements. Consequently, it generates ambiguous mappings in which entities such as “m6A” may be misclassified across multiple functional roles depending on their grammatical position, breaching the one-to-one correspondence principle. Most critically, its reliance on surface-level linguistic patterns rather than underlying relational structures hinders effective analogical alignment between biological and everyday conceptual domains.

The second theoretical foundation of MetaphorPrompt2 is the Causal Event Representation Theory by Gopnik and Wellman [24], which represents causal relationships in an Input-State → Process → Outcome-State format. Without equating LLM processing with child causal learning, we adopt this format as a structural template that forces the model to represent state transitions essential to biological causation, rather than collapsing causal chains into simple entity–action pairs.

For example, under this template, “Hypoxia induces HIF-1α stabilization by inhibiting proteasomal degradation” is parsed as “Hypoxia [low oxygen state] → inhibits proteasomal degradation [mechanistic process] → HIF-1α stabilization [accumulated protein state]”. This format preserves both the mechanistic process and the contextual states that define biological causation, supporting downstream KG construction.

In contrast, MetaphorPrompt employs a traditional agent-action-patient model that represented causal events as simple entity relationships. It could reduce the causal event in the previous example as “Hypoxia → activates → HIF-1α”, losing critical mechanistic information about the inhibitory process and the stability state changes. This representation failed to capture what Gopnik and Wellman identify as essential components of causal events: initial states, causal mechanisms, and resulting states.

These 2 theoretical foundations motivate the prompt architecture: SMT informs the design of the metaphor-aided extraction components (Context Identification, Named Entity Identification, Direct Interactions Identification, and Metaphor Development), while causal event representation informs the final Input-State -> Process -> Outcome-State extraction format. Together, they provide a theoretical rationale for the observed performance pattern, without implying direct evidence of analogical reasoning as an internal cognitive mechanism.

### MetaphorPrompt2: A structure and function-focused approach

**Fig. 1.**
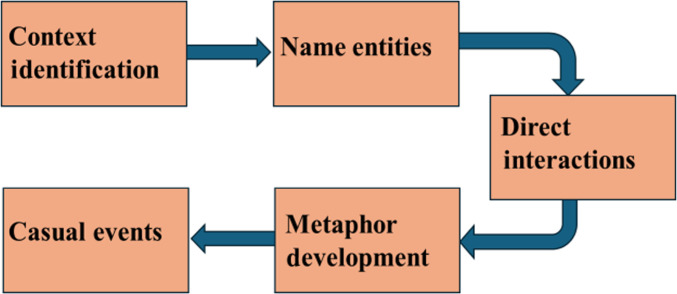
MetaphorPrompt2 flow chart illustrating the 5 main components and their interconnections.

To address the fundamental limitations of MetaphorPrompt, we developed the new architecture as illustrated in Fig. 1, showing various processing steps. Model and decoding details for all experiments are provided in the Experimental Design section to avoid duplication. Complete prompt templates for all 5 and a complete worked example showing the intermediate output of every component on a representative PubMed title is provided in Table S12.

#### Context identification

The prioritization of context identification as the initial step directly implements insights from cognitive theories of domain expertise. Research in cognitive psychology suggests that experts in specialized domains rely heavily on domain-specific semantic frames to constrain the interpretation of ambiguous information [27,28]. In MRP extraction, this principle is critical because biological terms often have multiple potential roles depending on experimental context.

For example, “diabetic mice” could theoretically be interpreted as either experimental subjects (context) or biological entities participating in regulatory pathways. By establishing context first, it creates what cognitive scientists term “semantic constraints” that prevent the system from generating metaphors that inappropriately map experimental conditions to causal agents in everyday scenarios.

#### Named Entity Identification

The Named Entity Identification component implements Gentner’s principle of systematic correspondence by classifying entities according to their functional roles in causal systems, either as initiators (entities that initiate an action or process), mediators (mediating entities that initiate and receive actions), or outcomes (outcomes of actions or processes), rather than their grammatical positions. This design choice reflects a key insight from SMT: Successful analogical reasoning depends on aligning functional relationships rather than surface features.

In biomedical texts, grammatical structure often obscures functional relationships. Consider the title “IGF2BP3 promotes colorectal cancer progression through EGFR stabilization in an m6A-dependent manner”. A grammar-based approach might classify “m6A” as an agent due to its position, but functional analysis reveals that it serves as a contextual modifier. SMT predicts that this misclassification will lead to poor analogical mappings because the functional-role misalignment prevents systematic correspondence between biological and metaphorical domains.

#### Direct interactions identification

The biggest challenge in extracting MRPs is the differentiation of direct interactions from indirect interactions that involve intermediary nodes in a long causal chain. Intermediary nodes are usually arranged in subclauses, making them grammatically distant from other nodes in the causal chain. This step requires LLMs to perform interaction identification through a combination of dependency parsing using the following prompt: “List all possible direct interactions between named entities. List the involved entities for each interaction.”

Unlike the previous method’s reliance on the arrangement of nouns in upstream and downstream patterns, the proposed method leverages LLMs’ dependency parsing to detect action words (regulates, activates, inhibits) and prepositions (by, through) that signify direct regulatory relationships. For example, in “Ubiquitination regulates proteasomal degradation”, the verb “regulates” is contextually linked to Ubiquitination (input) and proteasomal degradation (outcome).

This approach addresses the problem of determining mediators, which are introduced by prepositions like “through”, “via”, or “by”, following the preceding noun or verb. In titles like “m6A-mediated upregulation of HOXC10 promotes human hepatocellular carcinoma development through PTEN/AKT/mTOR signaling pathway”, the previous method consistently misidentified “PTEN/AKT/mTOR signaling pathway” as the target entity rather than a mediating process. In contrast, the current approach correctly identifies the signaling pathway as a mediator in the causal process.

This step essentially extracted the structural relations between named entities, which prepares LLMs for structurally aligned metaphors in the next step.

#### Metaphor development

After the previous 2 steps, the functional roles and structural relations between named entities have been extracted. In this step, identified relationships and functional roles are integrated for metaphor generation.

The prompt of this part is: “Develop a metaphor that accurately represents the roles, interactions, and relationships of biomedical entities, reflecting physiological processes, disease mechanisms, and medical interventions in maintaining or disrupting health.”

This prompt is designed to preserve the essential causal relationships identified in previous steps and to create close structural correspondences in the sense of Gentner [23].

For example, in the title “Ubiquitination Regulates the Proteasomal Degradation and Nuclear Translocation of the Fat Mass and Obesity-Associated (FTO) Protein”, the proposed method generates a metaphor that maintains close alignment with the functional roles and relationships:

“Ubiquitination acts as a traffic controller in the cell, directing 2 critical pathways: one that sends proteins to the recycling plant (proteasomal degradation) for breakdown, and another that moves proteins to the control center (nuclear translocation) of the cell.”

This metaphor preserves the essential causal relationships while making them accessible through familiar conceptual structures.

#### Causal event identification

After the metaphor has translated complex biological concepts into relatable real-world analogies, this final component implements an integrated causal link extraction mechanism leveraging the created metaphor:

“Extract all causal events based on the direct interactions in the form of input-action/process-outcome. Print each causal event in the format of input -> action/process -> outcome.”

### Workflow comparison between MetaphorPrompt and MetaphorPrompt2

The architectural differences between these prompts extend beyond their individual components to their overall workflow structures. The previous prompt design employed a linear, segmented process, where each step operated largely in isolation from subsequent steps. Context identification was treated as a separate step, followed by isolated phases for verb/preposition identification, causal event extraction, and standalone relational graph generation. This rigid sequencing created fragmented outputs due to limited cross-phase integration.

In contrast, the current method adopts a fluid, adaptive flow where context informs entity functional roles and structural relation identification precedes metaphor generation, facilitating the structural alignment in analogical reasoning. The reduction in procedural steps to the current 5-component structure reduces cognitive load and improve processing accuracy and reducing error propagation.

### Adjusting compound noun phrases

A substantial implementation challenge involves the treatment of compound noun phrases. MetaphorPrompt2 explicitly instructs the LLM to preserve compound noun phrases as single entities so that causal event outcomes are treated cohesively. For example, “proteasomal degradation” is maintained as a single mediator entity rather than being split into “proteasomal” and “degradation”.

However, this design can occasionally create mismatches when the ground-truth annotation lists individual nouns without modifiers, or when the LLM recasts an entity using a synonymous phrase. To address this issue, we introduce both a flexible node matching criterion (FNMC) and a strict node matching criterion (SNMC), which together provide upper- and lower-bound estimates of performances. These criteria are described in detail in the Node matching criteria section.

The compound noun phrase evaluation procedure was applied uniformly to MetaphorPrompt2, MetaphorPrompt, and the no-metaphor baseline under both flexible and strict evaluation settings.

### Testing dataset

The evaluation was conducted using 3 biomedical datasets to ensure a comprehensive assessment across different domain contexts and relation extraction challenges: the reguloGPT [12] benchmark dataset, the ADE (adverse drug events) corpus [26], and the BioInfer corpus [25].

#### Primary dataset: reguloGPT benchmark

The reguloGPT dataset [12] consists of 400 manually annotated PubMed titles focused on N6-methyladenosine (m6A) regulatory pathways. This dataset exhibits high entity diversity with 6 distinct molecular categories: Genes/Proteins, Gene Ontology (GO)/Pathway terms, m6A readers, m6A writers, erasers, and Other functional entities. The dataset suggests substantial causal interaction complexity with 1,558 nodes and 1,485 edges (1,312 unique nodes, 152 unique edge types), averaging 3.72 entity–relation pairs per title. After normalization, 1,241 unique nodes and 62 unique edges remain.

The dataset captures hierarchical m6A regulatory networks involving multi-step enzymatic cascades where writers install modifications, readers interpret them, and erasers remove them, creating complex interdependent pathway structures. It covers 24 different The Cancer Genome Atlas (TCGA) cancer types, providing broad pathological diversity within the specialized m6A research domain. Five expert annotators with computer science and biomedicine backgrounds ensured annotation quality.

#### Secondary dataset: ADE corpus

The ADE corpus [26] consists of 4,272 sentences extracted from 1,644 PubMed abstracts, containing a total of 6,821 manually annotated adverse drug events. Each entry represents a sentence containing drug-adverse effect relationships, with 5,063 drug–ADE pairs annotated as positive relationships and 1,758 as negative relationships. The ADE corpus focuses on medication-related relationships extracted from clinical discharge summaries in the MIMIC-III database. This dataset exhibits moderate entity diversity with 5 primary categories: Medications, Indications, Adverse Drug Events, Clinical Attributes (Route, Dosage, Duration, Frequency), and Patient Conditions.

The dataset captures medication-related adverse outcomes across diverse therapeutic areas, including cardiology, oncology, and psychiatry. The clinical language from real patient records introduces challenges such as abbreviated medical terminology, incomplete sentences, and temporal relationship inference.

#### Third dataset: BioInfer corpus

The BioInfer corpus [25] consists of 1,100 sentences extracted from biomedical research article abstracts, containing 2,662 named entities and 2,534 annotated relationships between genes, proteins, and RNA. The corpus provides comprehensive annotation for named entities, relationships, and syntactic dependencies, focusing on relationships between genes, proteins, and RNA. The dataset includes 1,000 positive relationship instances and 756 negative instances, with relationships annotated using predicates such as CONTROL, COREFER, and MODIFY that capture both direct regulatory relationships and coreference resolution.

The dataset covers interactions across 847 unique proteins, 524 genes, and 291 RNA molecules, representing diverse biological processes including transcriptional regulation, protein phosphorylation, and signal transduction pathways. Unlike other corpora that provide separate annotation types, BioInfer provides all key annotation types together for a single set of sentences, including entity relationships, syntactic structure, and coreference information.

### Evaluation metrics

We evaluated the proposed method using recall, precision, and F1 score for edge prediction. Because the main task is causal pathway extraction, the primary evaluation focuses on whether direct level-0 causal links are recovered correctly. Node-level performance is used to support error analysis, while edge-level F1 is used as the main summary metric.

#### Node matching criteria

We used both flexible and strict node matching criteria (NMC). Under FNMC, a node is counted as matched if either the predicted node contains the ground-truth node or the ground-truth node contains the predicted node. For example, “proteasomal degradation” matches “proteasomal degradation pathway”, and “FTO” matches “FTO protein”. This criterion accounts for cases where model outputs preserve biologically meaningful modifiers that are absent from the ground-truth annotation.

Under SNMC, a node is counted as matched only when the predicted node and ground-truth node match word for word. Both FNMC and SNMC were applied uniformly across all evaluated methods.

#### Edge matching criteria

Edge evaluation distinguishes between level-0 links (direct causal relationships) and level-1 links (derived or redundant relationships). Level-0 links represent the most granular and nonredundant causal interactions, such as “Protein A suppresses Protein C”, while level-1 links, such as “Protein A increases Protein B”, can be inferred from multiple level-0 links: “Protein A suppresses Protein C” and “Suppressed Protein C increases Protein B”. Level-1 links are not counted in the evaluation to avoid redundancy.

We evaluated edges under both flexible and strict criteria. Under the flexible edge matching criterion (FEMC), an edge is counted as detected when its source and target nodes match the corresponding ground-truth nodes under the selected node-matching criterion, regardless of predicate wording. This setting treats predicate differences as potential LLM recasting or synonym variation and provides a permissive upper-bound estimate.

Under the strict edge matching criterion (SEMC), an edge is counted as matched only when the source and target nodes match and the extracted predicate match the ground-truth predicate. Variations due to LLM recasting in synonyms will be counted as errors. SEMC therefore provides a very conservative lower-bound estimate.

Neither criterion should be interpreted as the exact true performance. FEMC may overestimate performance because it can treat genuine predicate errors as acceptable label variation. SEMC may grossly underestimate performance because it can penalize biologically equivalent predicate recasting by LLMs, such as “inhibits” versus “suppresses”, as mismatches. We therefore interpret FEMC and SEMC as approximate upper and lower bounds on extraction performance, with the true performance expected to lie between them.

In the main results, we use FNMC + FEMC as the default evaluation setting. We additionally report stricter settings, including SNMC + FEMC and FNMC + SEMC, in Tables S4 and S5 to show that the relative ranking of methods is preserved under stricter evaluation.

#### Testing metrics

A predicted edge is a true positive (TP) when both its source and target nodes match a level-0 ground-truth edge under the active node-matching and edge-matching criterion (FNMC/SNMC and FEMC/SEMC as defined in the Edge matching criteria section); otherwise it is a false positive (FP). For node-level error analysis, a predicted node is a TP when at least one ground-truth node satisfies the active matching criterion against it, and an FP otherwise.

Edge recall is computed as TP/(TP + FN), where FN (false negative) is the number of level-0 ground-truth edges not matched by any predicted edge. Edge precision is computed as TP/(TP + FP). Level-1 links are excluded before scoring to avoid double-counting relations derivable from level-0 links.

F1 is reported as the primary summary metric. F1 is the harmonic mean of precision and recall, F1 = 2·P·R/(P + R), and assigns equal weight to FPs and FNs. Equal weighting is appropriate here because both error types carry symmetric downstream consequences in MRP extraction: a missed link yields an incomplete causal chain in the resulting KG, while an incorrect link introduces an invalid causal edge. Full precision and recall values are reported in the supplementary tables.

Detailed error categories examining specific failure modes are described in the MRP element performance analysis section.

## Experimental Design

The experimental design aims at systematically assessing the contribution of metaphor-aided extraction, causal event identification, direct interaction identification, context identification, and functional-role named entity identification. All primary experiments were conducted using OpenAI’s GPT-4o (snapshot gpt-4o-2024-11-20) accessed via the OpenAI API. Decoding parameters were temperature = 0.1, top_p = 0.9, and max_tokens = 1,024. The reguloGPT benchmark was used as a common evaluation set to enable direct comparison. The benchmark does not provide a designated development split. Prompt templates were fixed before quantitative evaluation and applied without dataset-specific modification to BioInfer, ADE, and the cross-model experiments.

### Performance evaluation against baselines

To establish the effectiveness of the proposed method, we conducted a comparative performance evaluation against 2 baselines: a no-metaphor CoT baseline and the previous metaphor-based method. The no-metaphor baseline is derived directly from MetaphorPrompt2 by removing the metaphor-related component while retaining the remaining extraction steps. All 3 methods were freshly rerun using the same GPT-4o snapshot and identical decoding parameters; no baseline performance was copied from prior publications.

This experiment quantifies the performance gain attributable to the structure- and function-focused prompt architecture and the metaphor-aided extraction step. We report precision, recall, and F1 across 0-shot to 3-shot settings.

### Component ablation study

We conducted a component ablation study to quantify each component’s contribution and probe the structural and semantic dependencies within the metaphor framework.

The single-component ablations evaluate each of the 5 components individually. The 2-component ablations investigate synergistic effects and architectural dependencies—whether components depend on each other to function effectively. For example, removing both Context and Named Entity identification tests whether these 2 components work interdependently in establishing semantic constraints. The 2-component ablations also reveal non-additive degradation patterns, where combined removal causes a performance drop exceeding the sum of the individual drops, indicating that components enhance each other’s effectiveness through their interactions.

### Component degradation analysis

To further quantify the improvement of the current method over the previous method, we conducted an experiment in which key components of the new framework are replaced with those from the older version. The objective is to assess the performance degradation caused by reverting to older components in the previous metaphor-based prompts and to confirm the importance of the new refinements.

This experiment serves as a robustness check, ensuring that the changes in the proposed method are necessary for achieving superior performance.

### MRP element performance analysis

While the first 4 experiments are designed to measure the overall performance gain, in this experiment, we parse the performance gain under detailed categories, aiming to stratify the elements in MRPs that benefited most from the refinement:

1. Entity identification accuracy: We measure the accuracy in the identification of initiator and target entities (node A and node B) on MRPs.
2. Modifier misinterpretation: This aims to quantify incorrect handling of descriptive phrases such as “m6A-dependent manner”, where a grammar-first approach may misinterpret modifiers as primary entities.
3. Pathway splitting error: This refers to inappropriate fragmentation of compound pathway terms (e.g., “PTEN/AKT/mTOR signaling pathway” split into separate components).
4. Intermediate entity confusion: This aims to assess errors when mediating processes are misidentified as target nodes.
5. m6A contextual misinterpretation: This refers specifically to the misidentification of m6A’s role across various contexts as modifiers, processes, or entities.

Results related to item 1 is reported in Table 4, and the remaining 4 are reported in Table 5.

### Stability and bootstrap analysis setup

To address run-to-run variability inherent in LLM decoding even at low temperature, MetaphorPrompt2 was executed 4 independent times at each few-shot configuration (0-, 1-, 2-, and 3-shot) on the reguloGPT benchmark. Mean F1, precision, recall, and standard deviation across the 4 runs are reported per configuration.

To address robustness to test-set composition, we performed bootstrap resampling of the 400 reguloGPT titles with 1,000 bootstrap replicates per method–configuration pair. For each replicate, the same evaluation protocol (FNMC + FEMC) was applied, and the 95% CI was computed from the empirical distribution of F1 across replicates. Bootstrap CIs were computed for the no-metaphor baseline, MetaphorPrompt, and MetaphorPrompt2.

### Cross-backbone and model-recency setup

To evaluate whether the prompt architecture depends on a specific LLM backbone, we additionally ran the no-metaphor baseline, MetaphorPrompt, and MetaphorPrompt2 on 3 models beyond GPT-4o: a recent frontier model (GPT-5.4, snapshot gpt-5.4-2026-03-05) accessed via the OpenAI API, and 2 biomedical domain-tuned open-source models, Llama3-OpenBioLLM-70B and BioMistral-7B. The 2 open-source models were run at bfloat16 precision via local inference using their respective official chat templates. Decoding parameters across all 3 additional models matched the primary GPT-4o experiments (temperature = 0.1, top_p = 0.9, max_tokens = 1,024). The prompt template was held identical to the GPT-4o experiments; no model-specific tuning was performed.

GPT-5.4 was on all 3 datasets (reguloGPT, BioInfer, and ADE). The 2 open-source models were evaluated on reguloGPT to enable a per-shot comparison against GPT-4o.

### Qualitative metaphor analysis setup

To assess whether MetaphorPrompt2’s gains track with metaphor quality, we manually inspected paired metaphors on reguloGPT titles under FNMC + FEMC generated by MetaphorPrompt and MetaphorPrompt2. For each selected title, we recorded the source title, the generated metaphor from each method, the extracted edges, and the edge-level match status against ground truth. The full set of paired examples is provided in Files S2 and S3.

## Test Results

### Cross-dataset performance evaluation of MetaphorPrompt2

The performance evaluation suggests substantial improvements over baseline methods across 3 diverse biomedical datasets.

**Fig. 2.**
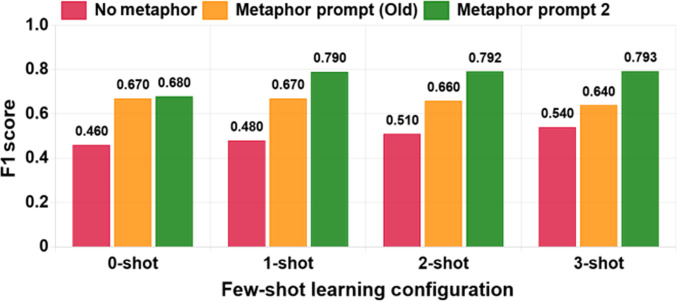
F1 score performance on reguloGPT dataset.

reguloGPT dataset performance. On the reguloGPT dataset (Fig. 2), the proposed method achieves the highest F1 performance across few-shot configurations. One-shot learning provides most of the observed gain, with F1 increasing from 0.68 at zero-shot to 0.79 at one-shot. At one-shot, the present method improves over the previous method by 12% and over the no-metaphor baseline by 31%.

**Fig. 3.**
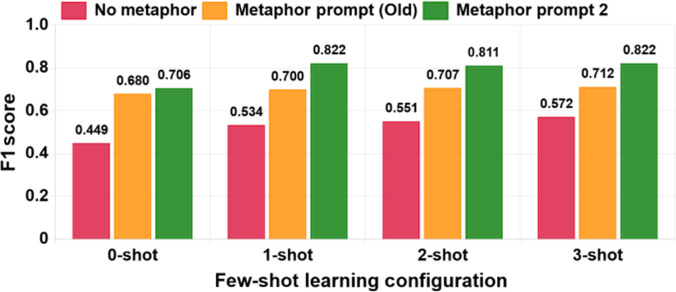
F1 score performance on BioInfer dataset.

BioInfer dataset performance. On the BioInfer dataset (Fig. 3), the proposed method achieved an F1 score of 0.822 with one-shot learning, representing a 12.2% improvement over MetaphorPrompt and a 28.8% improvement over the no-metaphor baseline. Performance remains stable across few-shot configurations, with F1 scores ranging from 0.811 to 0.822.

**Fig. 4.**
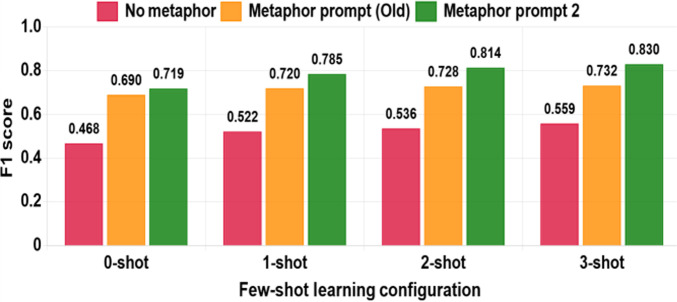
F1 score performance on ADE dataset.

ADE dataset performance. On the ADE dataset (Fig. 4), the proposed method achieved an F1 score of 0.790 at one-shot learning, representing a 6.5% improvement over MetaphorPrompt and a 26.8% over the no-metaphor baseline. Peak performance is achieved at 3-shot learning, where F1 reaches 0.830.

Across all 3 datasets, the current method consistently improves over the previous metaphor-based method and the no-metaphor baseline. The architectural gains exceed the small inter-dataset differences observed, supporting the view that the improvement reflects the prompt architecture rather than a single dataset-specific effect. Specifically, the absolute one-shot F1 difference between datasets is small (BioInfer − reguloGPT = 3.2%; ADE − reguloGPT = 0.5%), whereas the architectural improvement of MetaphorPrompt2 over MetaphorPrompt is 6.5% to 12.2%. The architectural effect is therefore consistently larger than the inter-dataset variation. We avoid attributing remaining inter-dataset variation such as those between reguloGPT and ADE to any single factor, because annotation conventions, terminology distributions, surface sentence properties, dataset exposure, and relational complexity may all contribute.

### Component ablation study

The component ablation study suggests that each component contributes to performance gains, as removing any single element leads to performance drops across all 3 datasets (Table 1).

**Table 1.** Component ablation test results on edge detection accuracy with one-shot ICL

Condition	reguloGPT F1 score	BioInfer F1 score	ADE F1 score
MetaphorPrompt2	0.7910	0.8220	0.8300
No Context	0.7067	0.7445	0.7525
No Name Entity	0.6931	0.7270	0.7355
With No Metaphor	0.6134	0.6425	0.6555
No Direct Interaction	0.5287	0.5560	0.5695
No Causal Link	0.5087	0.5355	0.5515

The results indicate that all components contribute to performance gains. Causal link identification produced the largest average degradation when removed followed by direct interaction identification, suggesting that these components are especially important for recovering MRPs.

The multi-component removal experiments show large performance drops across all 3 datasets (Table 2). Removing direct interaction and causal link components together caused the most severe degradation. These compound effects cannot be characterized as the summation of single-component effects, suggesting interdependencies among context, entity–role classification, direct interaction identification, metaphor development, and causal event extraction.

**Table 2.** Two-component removal study (reguloGPT, BioInfer, ADE datasets)

Components removed	reguloGPT F1	BioInfer F1	ADE F1
MetaphorPrompt2 (Complete)	0.7910	0.8200	0.8300
No Context + No Name Entity	0.6240	0.6650	0.6780
No Direct Interaction + No Causal Link	0.4180	0.4480	0.4680
With No Metaphor + No Context	0.5520	0.5890	0.6120
With No Metaphor + No Name Entity	0.5380	0.5720	0.5950
No Context + No Name Entity + With No Metaphor	0.4850	0.5180	0.5350

These findings support the hypothesis that the components of the proposed prompt work together to improve biomedical causal extraction.

### Component degradation study

The component degradation study suggests that the performance gains stem from genuine architectural advances, with each refined component contributing meaningfully when compared against its original counterpart (Table 3).

**Table 3.** Changing new metaphor with old metaphor condition

Component replaced with original version	reguloGPT F1 score	BioInfer F1 score	ADE F1 score
MetaphorPrompt2 (All Refined Components)	0.7910	0.8220	0.8300
Replace Context Identification	0.6920	0.7180	0.7250
Replace Named Entity Identification	0.6750	0.7020	0.7085
Replace Direct Interaction Identification	0.7120	0.7385	0.7465
Replace Metaphor Development	0.6520	0.6785	0.6855
Replace Causal Event Identification	0.6180	0.6435	0.6505

The degradation study supports the contribution of each refined component to overall performance, with the size of degradation tracking the centrality of each component to the new architecture.

The 2 components most directly tied to MetaphorPrompt2’s theoretical motivation—causal event representation and metaphor development—produced the largest performance drops when reverted. Replacing the Input-State → Process → Outcome-State encoding with the original agent-action-patient model gave the steepest decline (−21.7% average across datasets), supporting the utility of state-transition representation for biomedical causation. Replacing the refined metaphor development with the original grammar-based categorization gave the next largest drop (−17.5%), consistent with the shift from grammatical roles to functional classification being a primary source of improvement.

Functional-role named entity identification (−14.6%) and context-first identification (−12.6%) produced mid-range drops, indicating that they support but do not dominate the architectural advantage. Direct interaction identification produced the smallest drop (−10.1%), plausibly because the original upstream–downstream model already captured part of the relevant structure; the new placement of this step before metaphor generation still contributes measurably.

Notably, these element-wise degradations (10.1% to 21.7% on average) span a higher range than the gap between the full MetaphorPrompt2 and the full MetaphorPrompt across the 3 datasets at one-shot (6.5% to 12.2%). That replacing a single component can degrade performance more than reverting the entire architecture indicates that components within each architecture are mutually reinforcing rather than additive—a hybrid mixing one old component into the new framework is less coherent than either complete architecture on its own.

The relative ordering of the 5 drops is preserved across reguloGPT, BioInfer, and ADE, suggesting that the architectural refinements generalize beyond the m6A-focused training distribution. Broader evaluation on additional datasets remains needed to confirm this pattern.

### MRP element performance analysis

The MRP element performance analysis on the reguloGPT dataset reveals substantial reductions in missing MRP components across all error categories compared to the previous approach (Table 4).

**Table 4.** MRP element performance analysis (reguloGPT 1-shot)

Performance metric	MetaphorPrompt (FNMC)	MetaphorPrompt2 (FMNC)	MetaphorPrompt2 (SNMC)
Both nodes failed	7.70%	3.70%	5.60%
Only node A failed	13.30%	10.10%	12.20%
Only node B failed	14.70%	7.30%	10.30%

Table 4 shows that MetaphorPrompt2 reduces all 3 node-failure types relative to MetaphorPrompt—both-node failures and node B failures each drop by roughly half, while node A failures drop by about a quarter. The reductions persist under stricter matching criteria. Improvements are largest on m6A-related cases (72.2% for node A and 47.5% for node B), reflecting a design response to ambiguities observed in the previous method; comparable gains on BioInfer and ADE, which contain no m6A content, indicate that the architectural changes are not m6A-specific.

To isolate the mechanisms behind these improvements, we conducted error analyses categorizing failures into 4 types as shown in Table 5.

**Table 5.** Error category reduction between MetaphorPrompt and MetaphorPrompt2

Error category	MetaphorPrompt	MetaphorPrompt2	Error reduction (%)
Modifier misinterpretation	82 cases	35 cases	57.3%
Pathway splitting	40 cases	16 cases	60.0%
Intermediate entity confusion	125 cases	72 cases	42.4%
m6A role misinterpretation	171 cases	59 cases	65.5%

This analysis suggests improvements across all categories, with large reductions in m6A role misinterpretation, modifier misinterpretation, pathway splitting, and intermediate entity confusion.

### Stability and bootstrap confidence analysis

To address run-to-run variability, we evaluated the proposed method across 4 independent runs at each few-shot configuration on the reguloGPT dataset. Mean F1 scores were 0.684 (SD = 0.016) at 0-shot, 0.794 (SD = 0.018) at 1-shot, 0.798 (SD = 0.019) at 2-shot, and 0.796 (SD = 0.016) at 3-shot.

We also performed bootstrap resampling of the 400 reguloGPT titles with 1,000 replicates per configuration. The results on F1 score at one-shot are shown in Table 6.

**Table 6.** Bootstrap CIs at one-shot (reguloGPT)

Method	F1	95% CI
No-Metaphor baseline	0.497	[0.487, 0.506]
MetaphorPrompt	0.691	[0.684, 0.698]
MetaphorPrompt2	0.790	[0.783, 0.804]

These intervals do not overlap at one-shot, supporting that the architectural improvement is robust to test-set composition. Full stability and bootstrap results over multiple runs and 0 to 3 shots are provided in Tables S2 and S11.

### Cross-backbone and model-recency evaluation

To evaluate whether the new prompt design depends on a specific LLM backbone, we added experiments on biomedical domain-tuned models and a more recent frontier model GPT-5.4. The GPT-5.4 results are summarized in Table 7.

**Table 7.** GPT-5.4 F1 score across 3 datasets at one-shot

Method	reguloGPT	BioInfer	ADE
No-Metaphor baseline	0.596	0.562	0.582
MetaphorPrompt	0.734	0.729	0.748
MetaphorPrompt2	0.840	0.838	0.828

At one-shot, the present method improved over the previous method by 8.0% to 10.9% and over the no-metaphor baseline by 24.4% to 27.6%. Comparing to the 6.5% to 12.2%, and 26.8% to 31% gaps on GPT-4o results (see Figs. 2 to 4), GPT-5.4 largely maintained the performance gap.

Using the same prompt without modification, the proposed method achieved F1 scores from 0.680 to 0.779 across 0-shot to 3-shot settings on Llama3-OpenBioLLM-70B and 0.614 to 0.712 on BioMistral-7B, compared with 0.680 to 0.793 on GPT-4o over the same shot range.

These results, reported in detail in Tables S6 to S10, suggest that the prompt architecture transfers across model families and remains beneficial as backbone capability advances.

### Qualitative analysis of generated metaphors

We manually inspected metaphors on reguloGPT titles generated by MetaphorPrompt2 and MetaphorPrompt. Two patterns recur. MetaphorPrompt2’s metaphors are roughly half as long and stay closer to the title’s named entities; MetaphorPrompt frequently appends interpretive sentences not present in the source. More importantly, when a title contains a multi-token noun phrase that itself encodes a relation, MetaphorPrompt collapses the phrase into a single metaphorical actor, while MetaphorPrompt2 keeps the constituents distinct, and the downstream extraction inherits this choice.

For example, for the title “m6A deposition is regulated by PRMT1-mediated arginine methylation of METTL14”, MetaphorPrompt’s 45-word metaphor bound PRMT1 and arginine methylation into one “control technician” and recovered 0 of 3 edges; MetaphorPrompt2’s 18-word metaphor named PRMT1 as a distinct technician adjusting METTL14 and recovered all 3. On “FTO regulates ocular angiogenesis via m6A-YTHDF2-dependent mechanism”, MetaphorPrompt fused m6A and YTHDF2 into one “relay mechanism” (1 of 4 edges), while MetaphorPrompt2 named them separately (4 of 4).

These observations align with SMT: analogical inference relies on one-to-one structural correspondence, preserved most reliably when each title entity maps to a distinct actor. Metaphor length appears to be a proxy for this structural fidelity rather than its cause. The analysis is illustrative; a systematic large-scale evaluation is left to future work. Additional examples are provided in Files S2 and S3.

## Discussion

This research introduces MetaphorPrompt2, a structure- and function-focused, metaphor-aided extraction approach for MRP extraction. Across 3 datasets, the results suggest that the proposed method improves edge F1 relative to both the no-metaphor baseline and the previous MetaphorPrompt method while reducing several categories of causal link extraction errors.

Although the present method was motivated primarily by structure-mapping theory and causal event representation, the performance improvements are consistent with the theoretical principles motivating the new prompt design, providing a rationale for prioritizing functional roles, direct interactions, and state-transition encoding. However, the present results do not establish that the LLM is performing analogical transfer in Gentner’s cognitive-mechanism sense by internally executing structural alignment between source and target representations.

At the same time, the present study has not evaluated downstream utility. Database alignment against KEGG, Reactome, or SIGNOR could be useful as a utility-focused evaluation, but non-aligned triples may reflect extraction errors, curation lag, or incomplete database coverage. If reliable information about pathway database coverage and curation lag becomes available, database alignment could be incorporated as an additional evaluation.

While reguloGPT consists of PubMed titles, BioInfer and ADE provide sentence-level evaluation containing subordinate clauses, multi-entity dependencies, and clinical narrative vocabulary. The strong performance of the proposed method on these datasets (one-shot F1 of 0.822 on BioInfer and 0.830 on ADE) indicates that the pipeline handles within-sentence syntactic complexity beyond the title-only setting. Cross-sentence phenomena such as coreference, anaphora, and causal chains distributed across paragraphs are not represented in any of the 3 datasets and remain untested.

On ADE, the gain over MetaphorPrompt is the smallest of the 3 datasets (6.5 percentage points on GPT-4o, 8.0 percentage points on GPT-5.4), although the absolute F1 values on ADE are comparable to those on reguloGPT and BioInfer. The smaller gain may reflect factors such as task complexity, training exposure, or domain differences; a more detailed attribution analysis is left to future work.

The smaller gain on ADE should not be conflated with the substantially higher F1 reported on ADE by recent zero-shot benchmarks of biomedical relation extraction with LLMs [29]. Those benchmarks evaluate flat binary drug–effect classification, whereas our F1 reflects direct MRP extraction with the full causal pathway pipeline; the 2 sets of results are therefore not directly comparable.

In conclusion, MetaphorPrompt2 suggests that metaphor-aided extraction combined with functional-role classification and structural relationship extraction can improve MRP extraction across title- and sentence-level biomedical datasets. Several limitations frame the scope of these conclusions. The evaluation covers 3 datasets of varying linguistic complexity, but none contain multi-sentence causal chains, coreference, or anaphora distributed across paragraphs; extending the method to full abstracts and articles will require additional handling of these phenomena. Direct numerical comparison with contemporary biomedical relation extraction systems is limited by differences in task definition, supervision level, annotation scheme, and output structure, so we do not benchmark against them quantitatively. The pipeline-level evidence is consistent with SMT but does not constitute direct evidence that the LLM internally performs analogical reasoning. Finally, downstream utility—for example, in pathway database expansion, drug repurposing, or automated hypothesis generation—remains to be demonstrated.

## Future Work

Future work should evaluate MetaphorPrompt2 on PubMed abstracts and full-text articles, where multi-sentence causal reasoning, coreference, and anaphora present additional challenges. Future implementations may also combine the present method with retrieval-based grounding or provider-level structured-output enforcement, since these approaches are complementary to the proposed reasoning architecture. If reliable information about pathway database coverage and curation lag becomes available, database alignment could be considered as a utility-focused evaluation. A systematic analysis of metaphor structural fidelity, including successful and failed cases, would further clarify how generated metaphors contribute to extraction performance. In addition, direct mechanistic investigation—for example through attention analysis, activation patching, or behavioral probes designed to dissociate analogical mapping from generic prompt-structure effects—could help clarify whether the observed gains reflect genuine analogical reasoning by the model or improved prompt structuring.

## Data Availability

The data and related materials needed to evaluate the conclusions of this study will be made available in the GitHub repository: https://github.com/patelandpatel/MetaphorPrompt2. There are no restrictions on data availability.

## References

[1] Kanehisa M, Furumichi M, Tanabe M, Sato Y, Morishima K. KEGG: New perspectives on genomes, pathways, diseases and drugs. Nucleic Acids Res. 2017;45(D1):D353–D361. 27899662 10.1093/nar/gkw1092 PMC5210567

[2] Silva MC, Eugénio P, Faria D, Pesquita C. Ontologies and knowledge graphs in oncology research. Cancers. 2022;14(8):1906. 35454813 10.3390/cancers14081906 PMC9029532

[3] Bachman JA, Gyori BM, Sorger PK. Automated assembly of molecular mechanisms at scale from text mining and curated databases. Mol Syst Biol. 2023;19(5): Article MSB202211325. 10.15252/msb.202211325 PMC10167483 36938926

[4] Zhao D, Wang J, Zhang Y, Wang X, Lin H, Yang Z. Incorporating representation learning and multihead attention to improve biomedical cross-sentence n-ary relation extraction. BMC Bioinformatics. 2020;21(1):312. 32677883 10.1186/s12859-020-03629-9 PMC7364499

[5] Song L, Zhang Y, Wang Z, Gildea D. N-ary relation extraction using graph state LSTM. In: *Proceedings of the 2018 Conference on Empirical Methods in Natural Language Processing*. Brussels (Belgium): Association for Computational Linguistics; 2018. p. 2226–2235.

[6] Mi H, Thomas P. PANTHER pathway: An ontology-based pathway database coupled with data analysis tools. Methods Mol Biol. 2009;563:123–140. 19597783 10.1007/978-1-60761-175-2_7 PMC6608593

[7] OpenAI. GPT-4 Technical Report. arXiv. 2023. 10.48550/arXiv.2303.08774

[8] Wang B, Deng X, Sun H, Iteratively prompt pre-trained language models for chain of thought. In: *Proceedings of the 2022 Conference on Empirical Methods in Natural Language Processing*. Abu Dhabi (United Arab Emirates): Association for Computational Linguistics; 2022. p. 2714–2730.

[9] Lu Y, Bartolo M, Moore A. Riedel S, Stenetorp P. Fantastically ordered prompts and where to find them: Overcoming few-shot prompt order sensitivity. In: *Proceedings of the Annual Meeting of the Association for Computational Linguistics*. Dublin (Ireland): Association for Computational Linguistics; 2022. p. 8086–8098.

[10] Lewis P, Perez E, Piktus A, Petroni F, Karpukhin V, Goyal N, Küttler H, Lewis M, Yih Wt, Rocktäschel T, et al. Retrieval-augmented generation for knowledge-intensive NLP tasks. In: *Advances in Neural Information Processing Systems (NeurIPS)*. Red Hook (NY): Curran Associates Inc.; 2020. p. 9459–9474.

[11] Schick T, Dwivedi-Yu J, Dessì R, Raileanu R, Lomeli M, Zettlemoyer L, Cancedda N, Scialom T. Toolformer: Language models can teach themselves to use tools. In: *Advances in Neural Information Processing Systems (NeurIPS)*. Red Hook (NY): Curran Associates Inc.; 2023.

[12] X. Wu, Y. Zeng, A. Das, S. Jo, T. Zhang, P. Patel, J. Zhang, S.-J. Gao, D. Pratt, Y.-C. Chiu, Y. Huang. reguloGPT: Harnessing GPT for knowledge graph construction of molecular regulatory pathways. bioRxiv. 2024. 10.1101/2024.01.27.577521

[13] Wan G, Wu Y, Hu M, Chu Z, Li S. Bridging causal discovery and large language models: A comprehensive survey of integrative approaches and future directions. arXiv. 2024. 10.48550/arXiv.2402.11068

[14] Azam M, Chen Y, Arowolo MO, Liu H, Popescu M, Xu D. A comprehensive evaluation of large language models in mining gene interactions and pathway knowledge. bioRxiv. 2024. 10.1101/2024.01.21.576542 PMC11446478 39364206

[15] Patel P, Chiu YC, Hunag Y, Zhang J. MetaphorPrompt—An analogical reasoning approach for extracting causal links from biological text. In: *Proceedings of the 15th ACM International Conference on Bioinformatics, Computational Biology and Health Informatics*. New York (NY): ACM; 2024. p. 1–6.

[16] Yuan H, Hui SC, Zhang H. A structure-aware generative model for biomedical event extraction. arXiv. 2024. 10.48550/arXiv.2408.06583

[17] Zheng Y, Liu W, Zeng B, Feng Y, Du X, Zhou L, Li Y. Automating biomedical knowledge graph construction for context-aware scientific inference. bioRxiv. 2026. 10.64898/2026.01.14.699420

[18] Almeida T, Jonker RAA, Antunes R, Almeida JR, Matos S. Towards discovery: An end-to-end system for uncovering novel biomedical relations. *Database*. 2024;**2024**:baae057. 10.1093/database/baae057 PMC11240158 38994795

[19] Li J, Pan D, Yang Z, Sun Y, Lin H, Wang J. JTIS: Enhancing biomedical document-level relation extraction through joint training with intermediate steps. *Database*. 2024;**2024**:baae125. 10.1093/database/baae125 PMC11658465 39700498

[20] Dong X, Zhao D, Meng J, Guo B, Lin H. SyRACT: Zero-shot biomedical document-level relation extraction with synergistic RAG and CoT. Bioinformatics. 2025;41(7):btaf356. 40577808 10.1093/bioinformatics/btaf356 PMC12237500

[21] Shang Y, Guo Y, Hao S, Hong R. Biomedical relation extraction via adaptive document-relation cross-mapping and concept unique identifier. arXiv. 2025. 10.48550/arXiv.2501.05155.

[22] Hsu E, Roberts K. LLM-IE: A python package for biomedical generative information extraction with large language models. *JAMIA Open*. 2025;8(2):ooaf012. 10.1093/jamiaopen/ooaf012 PMC11901043 40078164

[23] Gentner D. Structure-mapping: A theoretical framework for analogy. Cogn Sci. 1983;7(2):155–170.

[24] Gopnik A, Wellman HM. Reconstructing constructivism: Causal models, Bayesian learning mechanisms, and the theory theory. Psychol Bull. 2012;138(6):1085. 22582739 10.1037/a0028044 PMC3422420

[25] Pyysalo S, Ginter F, Heimonen J, Björne J, Boberg J, Järvinen J, Salakoski T. BioInfer: A corpus for information extraction in the biomedical domain. BMC Bioinformatics. 2007;8(1):50. 17291334 10.1186/1471-2105-8-50 PMC1808065

[26] P Tafti A, Badger J, LaRose E, Shirzadi E, Mahnke A, Mayer J, Ye Z, Page D, Peissig P. Adverse drug event discovery using biomedical literature: A big data neural network adventure. JMIR Med Inform. 2017;5(4):e51. 29222076 10.2196/medinform.9170 PMC5741828

[27] Chi MTH, Feltovich PJ, Glaser R. Categorization and representation of physics problems by experts and novices. Cogn Sci. 1981;5(2):121–152.

[28] Ericsson KA, Lehmann AC. Expert and exceptional performance: Evidence of maximal adaptation to task constraints. Annu Rev Psychol. 1996;47(1):273–305. 15012483 10.1146/annurev.psych.47.1.273

[29] Brokman A, Ai X, Jiang Y, Gupta S, Kavuluru R. A benchmark for end-to-end zero-shot biomedical relation extraction with LLMs: Experiments with OpenAI models. In: *Proceedings of the Third Workshop for Artificial Intelligence for Scientific Publications (WASP@AACL)*. Mumbai (India) and virtual: Association for Computational Linguistics; 2025. p. 44–55.

