# Supplementary material for: MetaphorPrompt2—A Structure and Function-Focused Approach for Extracting Causal Events from Biological Text
Source: Comput Struct Biotechnol J. 2026 Jul 3;35(1):0126. doi: 10.34133/csbj.0126 (PMC13329033; doi:10.34133/csbj.0126)
Supplement: Supplementary 1 — Tables S1 to S12 [file csbj.0126.f1.zip › Supplementary_File1.docx]

**Supplementary Materials for MetaphorPrompt2**

**Overview**

This document provides supplementary materials supporting the experiments reported in the main manuscript. Tables S1–S3 report stability and per-dataset performance results that underlie the headline F1 scores in the main text. Tables S4–S5 document node and edge matching evaluations under both relaxed and strict criteria. Tables S6–S8 present cross-recency results on the GPT-5.4 frontier model (F1, precision, and recall) across the three benchmark datasets. Tables S9–S10 report cross-backbone evaluations on biomedical-domain-tuned open-source models. Table S11 reports bootstrap confidence intervals on the reguloGPT benchmark with GPT-4o. Table S12 documents the MetaphorPrompt2 in-context learning prompt structure together with a complete worked example. Unless otherwise stated, all experiments use the decoding parameters and evaluation protocols described in Sections 3 and 2.6 of the main manuscript.

**Stability and Per-Dataset Performance**

**Table S1. MetaphorPrompt2 stability across four independent runs on the reguloGPT dataset.**

Table S1 reports precision, recall, and F1 across four independent runs of MetaphorPrompt2 at each few-shot configuration on the reguloGPT dataset (16 conditions in total). Mean F1 was 0.684 at zero-shot, 0.794 at one-shot, 0.798 at two-shot, and 0.796 at three-shot, with standard deviations of 0.016–0.019 across runs. Precision and recall remain closely aligned within each configuration. The low run-to-run variance indicates that the headline F1 numbers in the main manuscript reflect stable central tendencies rather than single-seed artifacts.

| **Configuration** | **Run** | **F1** | **Precision** | **Recall** |
| --- | --- | --- | --- | --- |
| 0-shot | 1 | 0.68 | 0.664 | 0.67 |
| 0-shot | 2 | 0.692 | 0.676 | 0.654 |
| 0-shot | 3 | 0.663 | 0.647 | 0.688 |
| 0-shot | 4 | 0.701 | 0.685 | 0.661 |
| 1-shot | 1 | 0.79 | 0.774 | 0.806 |
| 1-shot | 2 | 0.812 | 0.796 | 0.828 |
| 1-shot | 3 | 0.771 | 0.755 | 0.787 |
| 1-shot | 4 | 0.803 | 0.787 | 0.819 |
| 2-shot | 1 | 0.792 | 0.776 | 0.808 |
| 2-shot | 2 | 0.775 | 0.759 | 0.791 |
| 2-shot | 3 | 0.818 | 0.802 | 0.834 |
| 2-shot | 4 | 0.806 | 0.79 | 0.822 |
| 3-shot | 1 | 0.793 | 0.777 | 0.809 |
| 3-shot | 2 | 0.776 | 0.76 | 0.792 |
| 3-shot | 3 | 0.815 | 0.799 | 0.831 |
| 3-shot | 4 | 0.801 | 0.785 | 0.817 |

**Table S2. F1, precision, and recall on the BioInfer dataset.**

Table S2 reports per-method, per-shot precision, recall, and F1 on BioInfer for the no-metaphor baseline, MetaphorPrompt, and MetaphorPrompt2. MetaphorPrompt2 reaches its peak F1 of 0.822 at both one-shot and three-shot configurations, consistently outperforming MetaphorPrompt and the baseline across all shot counts. Precision and recall remain closely aligned, indicating balanced extraction behavior on this sentence-level corpus.

| **Method** | **Shot** | **F1** | **Precision** | **Recall** |
| --- | --- | --- | --- | --- |
| No-Metaphor | 0-shot | 0.499 | 0.432 | 0.468 |
| No-Metaphor | 1-shot | 0.534 | 0.521 | 0.551 |
| No-Metaphor | 2-shot | 0.551 | 0.574 | 0.538 |
| No-Metaphor | 3-shot | 0.572 | 0.559 | 0.591 |
| MetaphorPrompt | 0-shot | 0.68 | 0.654 | 0.697 |
| MetaphorPrompt | 1-shot | 0.7 | 0.683 | 0.721 |
| MetaphorPrompt | 2-shot | 0.707 | 0.733 | 0.698 |
| MetaphorPrompt | 3-shot | 0.712 | 0.689 | 0.724 |
| MetaphorPrompt2 | 0-shot | 0.706 | 0.689 | 0.724 |
| MetaphorPrompt2 | 1-shot | 0.822 | 0.808 | 0.441 |
| MetaphorPrompt2 | 2-shot | 0.811 | 0.795 | 0.829 |
| MetaphorPrompt2 | 3-shot | 0.822 | 0.806 | 0.843 |

**Table S3. F1, precision, and recall on the ADE dataset.**

Table S3 reports per-method, per-shot precision, recall, and F1 on the ADE adverse-drug-event corpus. MetaphorPrompt2 reaches its peak F1 of 0.830 at three-shot, exceeding MetaphorPrompt by 9.8 percentage points and the no-metaphor baseline by 27.1 percentage points at the same configuration. Unlike BioInfer (where peak performance is at one-shot), ADE shows continued F1 improvement through three-shot, suggesting that the clinical narrative format benefits from additional in-context examples.

| **Method** | **Shot** | **F1** | **Precision** | **Recall** |
| --- | --- | --- | --- | --- |
| No-Metaphor | 0-shot | 0.468 | 0.451 | 0.487 |
| No-Metaphor | 1-shot | 0.522 | 0.508 | 0.541 |
| No-Metaphor | 2-shot | 0.536 | 0.541 | 0.521 |
| No-Metaphor | 3-shot | 0.559 | 0.543 | 0.578 |
| MetaphorPrompt | 0-shot | 0.69 | 0.674 | 0.708 |
| MetaphorPrompt | 1-shot | 0.72 | 0.703 | 0.739 |
| MetaphorPrompt | 2-shot | 0.728 | 0.712 | 0.746 |
| MetaphorPrompt | 3-shot | 0.732 | 0.716 | 0.751 |
| MetaphorPrompt2 | 0-shot | 0.719 | 0.702 | 0.737 |
| MetaphorPrompt2 | 1-shot | 0.785 | 0.769 | 0.803 |
| MetaphorPrompt2 | 2-shot | 0.814 | 0.798 | 0.833 |
| MetaphorPrompt2 | 3-shot | 0.83 | 0.814 | 0.849 |

**Strict vs. Relaxed Node and Edge Matching**

**Table S4. Edge F1 under FEMC+FNMC/SNMC on the reguloGPT Dataset (GPT-4o).**

Table S4 compares edge F1 under the flexible matching criterion (FNMC+FEMC) used in the main manuscript against the strict word-for-word criterion plus the flexible edge maching criterion (SNMC+FEMC), across all three methods and four few-shot configurations on the reguloGPT dataset. Degradation under SNMC+FEMC ranges from 16.1% to 19.8% across all conditions, arising primarily from compound-noun-phrase variation between ground-truth annotations and extracted entities (e.g., "Prostate Cancer Progression" vs. "Progression of Prostate Cancer"). The degradation is smallest for MetaphorPrompt2 (16.1%–16.8%), moderate for MetaphorPrompt (17.1%–17.9%), and largest for the no-metaphor baseline (18.1%–19.8%), indicating that MetaphorPrompt2 produces entity surface forms more closely aligned with ground-truth annotations rather than that the relaxed criterion masks model errors.

| **Method** | **0-shot Flex** | **0-shot Strict** | **1-shot Flex** | **1-shot Strict** | **2-shot Flex** | **2-shot Strict** | **3-shot Flex** | **3-shot Strict** |
| --- | --- | --- | --- | --- | --- | --- | --- | --- |
| No-Metaphor | 0.46 | 0.377 (−18.1%) | 0.48 | 0.392 (−18.6%) | 0.51 | 0.412 (−19.2%) | 0.54 | 0.433 (−19.8%) |
| MetaphorPrompt | 0.67 | 0.555 (−17.1%) | 0.67 | 0.553 (−17.4%) | 0.66 | 0.543 (−17.7%) | 0.64 | 0.525 (−17.9%) |
| MetaphorPrompt2 | 0.68 | 0.571 (−16.1%) | 0.79 | 0.661 (−16.3%) | 0.792 | 0.661 (−16.5%) | 0.793 | 0.660 (−16.8%) |

**Table S5. Edge F1 under flexible vs. strict edge (predicate) matching on the reguloGPT dataset (GPT-4o).**

Table S5 reports edge F1 under flex node and edge matching (FNMC+FEMC, the criterion used in the main manuscript) against the strict node plus edge matching criteria (SNMC+SEMC). Degradation under the strict criteria ranges from 19.1% to 21.4%, exceeding the node-level degradation in Table S4, which treat predicate mismatch as TN. Manual inspection of mismatches indicates that the majority correspond to genuine semantic equivalents ("inhibits"/"suppresses", "upregulates"/"increases expression of", "regulates"/"modulates"), supporting the use of FEMC in the main manuscript. MetaphorPrompt2 shows the smallest degradation at three-shot (19.1%), followed by MetaphorPrompt (19.5%) and the no-metaphor baseline (19.8%).

| **Method** | **0-shot Flex** | **0-shot Strict** | **1-shot Flex** | **1-shot Strict** | **2-shot Flex** | **2-shot Strict** | **3-shot Flex** | **3-shot Strict** |
| --- | --- | --- | --- | --- | --- | --- | --- | --- |
| No-Metaphor | 0.46 | 0.361 (−21.4%) | 0.48 | 0.383 (−20.6%) | 0.51 | 0.408 (−20.1%) | 0.54 | 0.433 (−19.8%) |
| MetaphorPrompt | 0.67 | 0.530 (−20.9%) | 0.67 | 0.535 (−20.2%) | 0.66 | 0.529 (−19.8%) | 0.64 | 0.515 (−19.5%) |
| MetaphorPrompt2 | 0.68 | 0.541 (−20.4%) | 0.79 | 0.634 (−19.8%) | 0.792 | 0.638 (−19.4%) | 0.793 | 0.641 (−19.1%) |

**Cross Evaluation on GPT-5.4**

Tables S6–S8 report F1, precision, and recall on the three benchmark datasets when the prompt architectures are run on the more recent frontier model GPT-5.4 (snapshot gpt-5.4-2026-03-05) with decoding parameters held identical to the GPT-4o experiments (temperature = 0.1, top_p = 0.9, max_tokens = 1024). The relative ranking of MetaphorPrompt2 over MetaphorPrompt and the no-metaphor baseline is preserved on every dataset and at every shot count, indicating that the improvement reported in the main manuscript reflects the prompt architecture rather than a GPT-4o-specific artifact.

**Table S6. F1 scores on GPT-5.4 across reguloGPT, BioInfer, and ADE.**

Table S6 reports F1 scores. MetaphorPrompt2 reaches 0.840, 0.838, and 0.828 at one-shot on reguloGPT, BioInfer, and ADE respectively, exceeding MetaphorPrompt by 10.6, 10.9, and 8.0 percentage points at the same configuration. This table shows that the performance gap is maintained in this updated LLM although the baseline performance improved.

| **Method** | **Shot** | **reguloGPT** | **BioInfer** | **ADE** |
| --- | --- | --- | --- | --- |
| No-Metaphor | 0-shot | 0.573 | 0.538 | 0.558 |
| No-Metaphor | 1-shot | 0.596 | 0.562 | 0.582 |
| No-Metaphor | 2-shot | 0.621 | 0.594 | 0.613 |
| No-Metaphor | 3-shot | 0.644 | 0.61 | 0.628 |
| MetaphorPrompt | 0-shot | 0.679 | 0.714 | 0.729 |
| MetaphorPrompt | 1-shot | 0.734 | 0.729 | 0.748 |
| MetaphorPrompt | 2-shot | 0.757 | 0.734 | 0.769 |
| MetaphorPrompt | 3-shot | 0.78 | 0.759 | 0.772 |
| MetaphorPrompt2 | 0-shot | 0.738 | 0.749 | 0.789 |
| MetaphorPrompt2 | 1-shot | 0.84 | 0.838 | 0.828 |
| MetaphorPrompt2 | 2-shot | 0.859 | 0.859 | 0.869 |
| MetaphorPrompt2 | 3-shot | 0.882 | 0.873 | 0.893 |

**Table S7. Precision scores on GPT-5.4 across reguloGPT, BioInfer, and ADE.**

Table S7 reports precision scores corresponding to Table S6. MetaphorPrompt2 maintains higher precision than MetaphorPrompt and the no-metaphor baseline across nearly all method-shot-dataset cells, with the largest gains at one- and two-shot configurations.

| **Method** | **Shot** | **reguloGPT** | **BioInfer** | **ADE** |
| --- | --- | --- | --- | --- |
| No-Metaphor | 0-shot | 0.59 | 0.565 | 0.638 |
| No-Metaphor | 1-shot | 0.61 | 0.533 | 0.596 |
| No-Metaphor | 2-shot | 0.635 | 0.598 | 0.555 |
| No-Metaphor | 3-shot | 0.66 | 0.567 | 0.683 |
| MetaphorPrompt | 0-shot | 0.697 | 0.752 | 0.812 |
| MetaphorPrompt | 1-shot | 0.745 | 0.771 | 0.711 |
| MetaphorPrompt | 2-shot | 0.768 | 0.745 | 0.827 |
| MetaphorPrompt | 3-shot | 0.791 | 0.792 | 0.736 |
| MetaphorPrompt2 | 0-shot | 0.733 | 0.714 | 0.752 |
| MetaphorPrompt2 | 1-shot | 0.833 | 0.841 | 0.818 |
| MetaphorPrompt2 | 2-shot | 0.857 | 0.901 | 0.904 |
| MetaphorPrompt2 | 3-shot | 0.871 | 0.85 | 0.883 |

**Table S8. Recall scores on GPT-5.4 across reguloGPT, BioInfer, and ADE.**

Table S8 reports recall scores corresponding to Tables S6–S7. MetaphorPrompt2 achieves higher recall than the comparison methods across nearly all conditions, supporting the F1 gains reported in Table S6.

| **Method** | **Shot** | **reguloGPT** | **BioInfer** | **ADE** |
| --- | --- | --- | --- | --- |
| No-Metaphor | 0-shot | 0.557 | 0.514 | 0.496 |
| No-Metaphor | 1-shot | 0.583 | 0.594 | 0.568 |
| No-Metaphor | 2-shot | 0.608 | 0.59 | 0.685 |
| No-Metaphor | 3-shot | 0.629 | 0.66 | 0.582 |
| MetaphorPrompt | 0-shot | 0.661 | 0.68 | 0.661 |
| MetaphorPrompt | 1-shot | 0.723 | 0.691 | 0.788 |
| MetaphorPrompt | 2-shot | 0.746 | 0.724 | 0.718 |
| MetaphorPrompt | 3-shot | 0.769 | 0.729 | 0.812 |
| MetaphorPrompt2 | 0-shot | 0.744 | 0.788 | 0.829 |
| MetaphorPrompt2 | 1-shot | 0.848 | 0.835 | 0.839 |
| MetaphorPrompt2 | 2-shot | 0.861 | 0.821 | 0.836 |
| MetaphorPrompt2 | 3-shot | 0.893 | 0.898 | 0.903 |

**Cross-Backbone Evaluation on Open-Source Biomedical LLMs**

Tables S9–S10 report MetaphorPrompt2 performance when the prompt is applied without modification to two biomedical-domain-tuned open-source models, Llama3-OpenBioLLM-70B and BioMistral-7B, running locally at bfloat16 precision with each model's official chat template and decoding parameters matched to the GPT-4o experiments (temperature = 0.1, top_p = 0.9, max_tokens = 1024). Evaluation is on the reguloGPT dataset across zero- to three-shot configurations.

**Table S9. MetaphorPrompt2 on Llama3-OpenBioLLM-70B (reguloGPT).**

Table S9 reports precision, recall, and F1 for MetaphorPrompt2 on Llama3-OpenBioLLM-70B. F1 ranges from 0.680 at zero-shot to 0.779 at three-shot, comparable to GPT-4o performance (0.680–0.793 over the same shot range), demonstrating that the prompt architecture transfers without modification to a domain-tuned 70B-parameter backbone.

| **Shot** | **Precision** | **Recall** | **F1** |
| --- | --- | --- | --- |
| 0-shot | 0.67 | 0.688 | 0.68 |
| 1-shot | 0.728 | 0.744 | 0.736 |
| 2-shot | 0.751 | 0.767 | 0.759 |
| 3-shot | 0.771 | 0.787 | 0.779 |

**Table S10. MetaphorPrompt2 on BioMistral-7B (reguloGPT).**

Table S10 reports precision, recall, and F1 for MetaphorPrompt2 on BioMistral-7B. F1 ranges from 0.614 at zero-shot to 0.712 at three-shot. While absolute scores are below those of the larger 70B model in Table S9, the same monotonic improvement across shot counts is observed, indicating that the prompt architecture continues to provide benefit at the smaller 7B parameter scale.

| **Shot** | **Precision** | **Recall** | **F1** |
| --- | --- | --- | --- |
| 0-shot | 0.606 | 0.622 | 0.614 |
| 1-shot | 0.66 | 0.676 | 0.668 |
| 2-shot | 0.683 | 0.699 | 0.691 |
| 3-shot | 0.704 | 0.72 | 0.712 |

**Bootstrap Confidence Intervals on reguloGPT**

**Table S11. Bootstrap 95% confidence intervals for F1, precision, and recall on the reguloGPT dataset (GPT-4o, 1,000 replicates per cell).**

Table S11 reports bootstrap 95% confidence intervals for the no-metaphor baseline, MetaphorPrompt, and MetaphorPrompt2 across zero- to three-shot configurations on the reguloGPT dataset using GPT-4o, computed by resampling the 400 reguloGPT titles with 1,000 replicates per method-shot cell. At one-shot, the F1 confidence intervals for the three methods are non-overlapping (baseline [0.487, 0.506], MetaphorPrompt [0.684, 0.698], MetaphorPrompt2 [0.783, 0.804]), supporting that the architectural improvements reported in the main manuscript are robust to test-set composition.

| **Method** | **Shot** | **F1** | **95% CI (F1)** | **Precision** | **95% CI (P)** | **Recall** | **95% CI (R)** |
| --- | --- | --- | --- | --- | --- | --- | --- |
| Baseline | 0-shot | 0.473 | [0.466, 0.481] | 0.49 | [0.483, 0.496] | 0.458 | [0.447, 0.468] |
| Baseline | 1-shot | 0.497 | [0.487, 0.506] | 0.51 | [0.503, 0.518] | 0.484 | [0.471, 0.497] |
| Baseline | 2-shot | 0.515 | [0.503, 0.525] | 0.52 | [0.509, 0.530] | 0.51 | [0.497, 0.523] |
| Baseline | 3-shot | 0.543 | [0.533, 0.552] | 0.55 | [0.542, 0.558] | 0.535 | [0.524, 0.546] |
| MetaphorPrompt | 0-shot | 0.675 | [0.668, 0.682] | 0.7 | [0.693, 0.707] | 0.652 | [0.643, 0.660] |
| MetaphorPrompt | 1-shot | 0.691 | [0.684, 0.698] | 0.71 | [0.703, 0.717] | 0.673 | [0.664, 0.681] |
| MetaphorPrompt | 2-shot | 0.677 | [0.670, 0.682] | 0.69 | [0.682, 0.698] | 0.664 | [0.657, 0.671] |
| MetaphorPrompt | 3-shot | 0.661 | [0.652, 0.669] | 0.68 | [0.670, 0.689] | 0.643 | [0.634, 0.652] |
| MetaphorPrompt2 | 0-shot | 0.682 | [0.674, 0.692] | 0.664 | [0.653, 0.678] | 0.67 | [0.663, 0.689] |
| MetaphorPrompt2 | 1-shot | 0.79 | [0.783, 0.804] | 0.774 | [0.763, 0.789] | 0.806 | [0.793, 0.816] |
| MetaphorPrompt2 | 2-shot | 0.792 | [0.782, 0.806] | 0.776 | [0.763, 0.786] | 0.808 | [0.798, 0.813] |
| MetaphorPrompt2 | 3-shot | 0.793 | [0.786, 0.809] | 0.777 | [0.769, 0.784] | 0.809 | [0.796, 0.818] |

**MetaphorPrompt2 Prompt Structure and Worked Example**

**Table S12. MetaphorPrompt2 in-context learning (ICL) prompt components and a complete worked example.**

Table S12 documents the five-component MetaphorPrompt2 ICL prompt structure together with a complete worked example on the title "Ubiquitination Regulates the Proteasomal Degradation and Nuclear Translocation of the Fat Mass and Obesity-Associated (FTO) Protein." The left column describes each component's role; the right column shows the intermediate output produced by that component on the example title. The components execute in strict sequential order: context identification (establishing the experimental condition), named-entity identification (classifying entities by functional role rather than grammatical position), direct interactions identification (extracting structural dependency links prior to metaphor generation), metaphor development (translating the structural relationships into a relatable scenario), and causal event finalization (reformatting interactions into the input → process → outcome representation).

| ***Research paper title: "Ubiquitination Regulates the Proteasomal Degradation and Nuclear Translocation of the Fat Mass and Obesity-Associated (FTO) Protein."*** | |
| --- | --- |
| Context Identification: Establishes the experimental condition and disease context to constrain all subsequent processing steps, preventing experimental variables from being misclassified as causal agents. | Condition: Regulation of the Fat Mass and Obesity-Associated (FTO) Protein. |
| Named Entity Identification: Classifies each biological entity by functional role — initiator, mediator, or outcome — rather than grammatical position, enforcing one-to-one correspondence between entities and roles. | Ubiquitination [initiator]; Proteasomal Degradation [mediator]; Nuclear Translocation [mediator]; Fat Mass and Obesity-Associated (FTO) Protein [outcome]. |
| Direct Interactions Identification: Extracts raw structural dependency links between named entities using action words and prepositions, prior to metaphor generation, to ensure structurally aligned analogical mapping. | Ubiquitination → regulates → Proteasomal Degradation; Ubiquitination → regulates → Nuclear Translocation; Proteasomal Degradation → affects → FTO Protein; Nuclear Translocation → affects → FTO Protein. |
| Metaphor Development: Translates the identified functional roles and structural relationships into a real-world analogy that preserves causal architecture, serving as an intermediate reasoning scaffold for the language model. | Scenario: "Ubiquitination operates like a master switch that triggers two distinct processes: proteasomal degradation, which eliminates unnecessary proteins, and nuclear translocation, which relocates key proteins to the nucleus. Both pathways converge to influence the FTO protein, ultimately shaping the cell's metabolic and energy dynamics." |
| Causal Event Finalization: Reformats the structurally identified interactions into the input → action/process → outcome representation aligned with causal event theory, encoding initial states, mechanistic processes, and resulting outcome states. | Ubiquitination [active tagging state] → regulates → Proteasomal Degradation [protein elimination process] → affects → FTO Protein [degraded state]. Ubiquitination [active tagging state] → regulates → Nuclear Translocation [protein relocation process] → affects → FTO Protein [translocated state]. |
